# Short-term supplementation of celecoxib-shifted butyrate production on a simulated model of the gut microbial ecosystem and ameliorated in vitro inflammation

**DOI:** 10.1038/s41522-020-0119-0

**Published:** 2020-02-19

**Authors:** Emma Hernandez-Sanabria, Evelien Heiremans, Marta Calatayud Arroyo, Ruben Props, Laurent Leclercq, Jan Snoeys, Tom Van de Wiele

**Affiliations:** 1 Center for Microbial Ecology and Technology (CMET), Ghent University, Coupure Links 653, 9000 Ghent, Belgium; 2 Janssen Research & Development, A Division of Janssen Pharmaceutica NV, Turnhoutseweg 30, Beerse, Antwerp 2340 Belgium

**Keywords:** Cellular microbiology, Applied microbiology, Microbiome

## Abstract

Celecoxib has been effective in the prevention and treatment of chronic inflammatory disorders through inhibition of altered cyclooxygenase-2 (COX-2) pathways. Despite the benefits, continuous administration may increase risk of cardiovascular events. Understanding microbiome-drug-host interactions is fundamental for improving drug disposition and safety responses of colon-targeted formulations, but little information is available on the bidirectional interaction between individual microbiomes and celecoxib. Here, we conducted in vitro batch incubations of human faecal microbiota to obtain a mechanistic proof-of-concept of the short-term impact of celecoxib on activity and composition of colon bacterial communities. Celecoxib-exposed microbiota shifted metabolic activity and community composition, whereas total transcriptionally active bacterial population was not significantly changed. Butyrate production decreased by 50% in a donor-dependent manner, suggesting that celecoxib impacts in vitro fermentation. Microbiota-derived acetate has been associated with inhibition of cancer markers and our results suggest uptake of acetate for bacterial functions when celecoxib was supplied, which potentially favoured bacterial competition for acetyl-CoA. We further assessed whether colon microbiota modulates anti-inflammatory efficacy of celecoxib using a simplified inflammation model, and a novel in vitro simulation of the enterohepatic metabolism. Celecoxib was responsible for only 5% of the variance in bacterial community composition but celecoxib-exposed microbiota preserved barrier function and decreased concentrations of IL-8 and CXCL16 in a donor-dependent manner in our two models simulating gut inflammatory milieu. Our results suggest that celecoxib-microbiome-host interactions may not only elicit adaptations in community composition but also in microbiota functionality, and these may need to be considered for guaranteeing efficient COX-2 inhibition.

## Introduction

Prostaglandin E_2_ (PGE_2_) is a key mucosal inflammatory mediator produced when membrane phospholipids are metabolized by cyclooxygenase (COX) enzymes. Increased synthesis of prostaglandins is a feature of chronic inflammation^1^ and has been proposed to promote tumorigenesis in the colon.^2^ Previous research showed that COX-2 expression correlates to invasiveness, prognosis, and survival in some cancers,^3^ such as colorectal cancer (CRC),^4^ causing proliferation, enhanced angiogenesis and apoptosis suppression.^5^ In this way, COX-2 inhibitors such as celecoxib (CX) are promising chemopreventive agents for chronic pain, inflammatory and mood disorders^6^ and CRC.^7^ Additionally, CX has demonstrated anti-inflammatory and analgesic effects, with lower incidence of upper gastrointestinal ulceration and complications during single and multiple administration for inflammatory conditions.^8^ Despite these benefits, their long-term use may shift cardiovascular homeostasis, inhibiting biosynthesis of vascular COX-2-dependent prostacyclin (PGI_2_)^9^ increasing risk for cardiovascular events,^10^ renal ischaemia,^11^ impaired fracture healing and peripheral oedema.^12^

Advances in omics techniques have provided insights on the role of the gut microbiome in cancer development.^13^ Currently, the driver-passenger model suggests that intestinal “driver” bacteria contribute to initiate CRC by damaging epithelial DNA.^14^ This microenvironment alteration impairs barrier function and favours opportunistic “passenger” bacteria, placing selective pressure on the microbiome.^14^

When colon-targeted drugs are orally administered, they encounter the gut microbiota before reaching host tissues. Microbial drug metabolism may impact drug stability and activity, influencing toxicity and inflammation on the host.^15^ Conversely, drugs may also alter the gut microbiota, modulating community structure, and potentially resulting in dysbiosis.^16^ Understanding microbiome-drug-host interactions is crucial to assess the potential impact on ADME and safety responses of intestinal targets. Although the gut microbiome plays an enormous role in patient health, and in pharmacokinetic and pharmacodynamic response,^17^ pharmaceutical companies only recently started to explore the microbiome as potential contributor to drug outcomes. Similarly, regulatory agencies are not currently considering gut bacteria within the official evaluations before approval of colon-targeted drugs. Besides the microbial modulation of drug bioavailability and pharmacokinetics,^15^ low solubility and small aqueous volumes in the colon may hinder drug efficacy.^18^ As interest in developing novel colon-targeted NSAID delivery systems has increased, insight into the presystemic biotransformation by the gut microbiota is fundamental. In vitro studies showed that bacteria can metabolize CX,^19^ potentially jeopardizing its anti-inflammatory effects. Additionally, whether CX presence can modulate the host-microbiome crosstalk remains unresolved. Prior reports correlating gut microbiome changes with CX treatment relied on DNA amplicon sequencing.^20^ As the presence of dead bacterial cells may obscure the interpretation of microbiome data, we analysed the bacterial community composition based on cDNA, as a proxy for the metabolically active community.

CX can be absorbed throughout the GI tract, but 85% of the dose is eliminated through urine and faeces.^8^ Overall systemic absorption of poorly soluble drugs may be improved with colon-delivery systems,^21^ because colonic transit times (17–24 h)^21,22^ are longer compared to those of the small intestine (3–4 h). Thus, we simulated delivery of a clinical dose of CX^8,23,24^ in a proximal colon-targeted formulation. We aimed at screening short-term bidirectional interactions between CX and diversity, composition and functionality of the human microbiome using an in vitro model of the proximal colon, and at further elucidating the ultimate impact of the microbiota on the anti-inflammatory efficacy of CX.

## Results

### CX impacts bacterial fermentative metabolism in a donor-dependent manner

CX-microbiota interactions were evaluated under proximal colon conditions (pH 5.6–5.9, 37 °C, 16 h of transit time^25–27^), as absorption of poorly soluble drugs may be improved using colon-delivery systems.^21^ Short-chain fatty acids (SCFAs) were used to monitor functional activities of the colon communities following exposure to CX or its vehicle (PEG). Measurements in volunteers 1 and 2 were missed at the time 0 h (Table 1). Total SCFA production from separate faecal microbiota incubations of eight different individuals was significantly lower for CX incubations compared to those with PEG (*P* < 0.05, Table 1b), but pH remained stable throughout the experiment (Supplementary Table 1). While the 60:20:20 ratio of the three major SCFA (acetate, propionate, and butyrate) was consistent across donors at the initial time point (60:16:7, Table 1a), a shift was evident at the end of the incubation (line 15, Supplementary Table 1). Only the mean relative proportion of acetate tended to be higher with CX (*P* = 0.06, Supplementary Table 1), while both relative and absolute concentrations of butyrate and propionate per individual were higher with PEG compared to those with CX (Table 1b). In fact, CX significantly lowered mean butyrate after 16 h (*P* < 0.05, Supplementary Table 1), except in donors 1, 4 and 8 (Table 1b). Total active bacterial load remained unchanged when CX was supplemented (Supplementary Table 2), but large variation was observed (Supplementary Table 1). These findings might indicate that bacteria either alter their metabolic activity when exposed to CX or that changes in bacterial composition lead to shifts in fermentation profiles.

**Table 1:** Relative percentages of the three major short-chain fatty acids (SCFA) derived from microbial fermentation, following supplementation with either celecoxib (CX) or the carrier (PEG) at the (A) beginning of the incubation (0 h, *n* = 6).

SCFA (%)	Ratio found in colon and stool^83^	Donor 3		Donor 4		Donor 5		Donor 6		Donor 7		Donor 8					
		CX	PEG	CX	PEG	CX	PEG	CX	PEG	CX	PEG	CX		PEG			
(A)																	
Acetate	60	60.02	65.71	35.45	39.30	63.90	65.32	59.25	63.42	40.88	41.84	49.98		53.97			
Propionate	20	23.20	21.64	14.84	14.46	12.63	12.48	12.83	11.70	14.20	13.97	10.61		10.81			
Butyrate	20	15.49	12.65	10.17	9.48	15.85	15.52	8.20	5.84	6.92	7.20	6.23		6.15			

SCFA (%)	Ratio found in colon and stool^83^	Donor 1		Donor 2		Donor 3		Donor 4		Donor 5		Donor 6		Donor 7		Donor 8	
		CX	PEG	CX	PEG	CX	PEG	CX	PEG	CX	PEG	CX	PEG	CX	PEG	CX	PEG
(B)																	
Acetate	60	49.41	47.15	74.64	62.51**	22.46	28.97	33.32	34.76	73.82	57.87***	84.89	63.64***	68.12	67.87	73.99	69.94
Propionate	20	17.30	18.05	22.40	18.27	21.01	18.89	6.90	9.06	14.96	16.13	12.58	18.93**	16.14	13.88	14.18	13.48
Butyrate	20	15.13	17.15	2.27	17.89***	4.83	11.76***	7.51	7.89	6.04	19.48***	2.46	13.01***	8.15	10.29**	7.66	8.07

Means between treatments were not significantly different and samples from donors 1 and 2 were missed at this time point. Quantification of relative SCFA percentage was completed again when incubation was finalized after 16 h (B). *N* = 8; **P < 0.05; ***P < 0.0001.

### Bacterial abundance remains stable, but individual microbiome diversity and composition shifted following CX supplementation

**Fig. 1:**
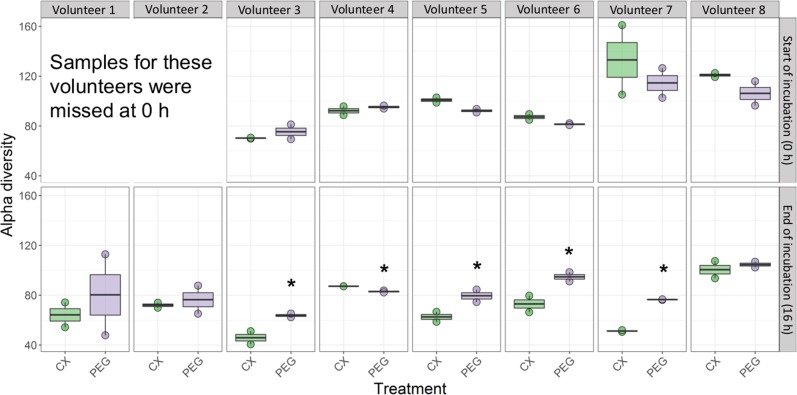
Inverse Simpson index (*D*_2_) was used to assess alpha diversity at the start (0 h) and at the end of the incubation (16 h post treatment) on each volunteer. Horizontal bars indicate means and distribution of each data point. Alpha diversity is indicated in green boxes for celecoxib treatment (CX), and in purple when the control treatment with the vehicle (PEG) was supplied. *P < 0.05.

**Fig. 2:**
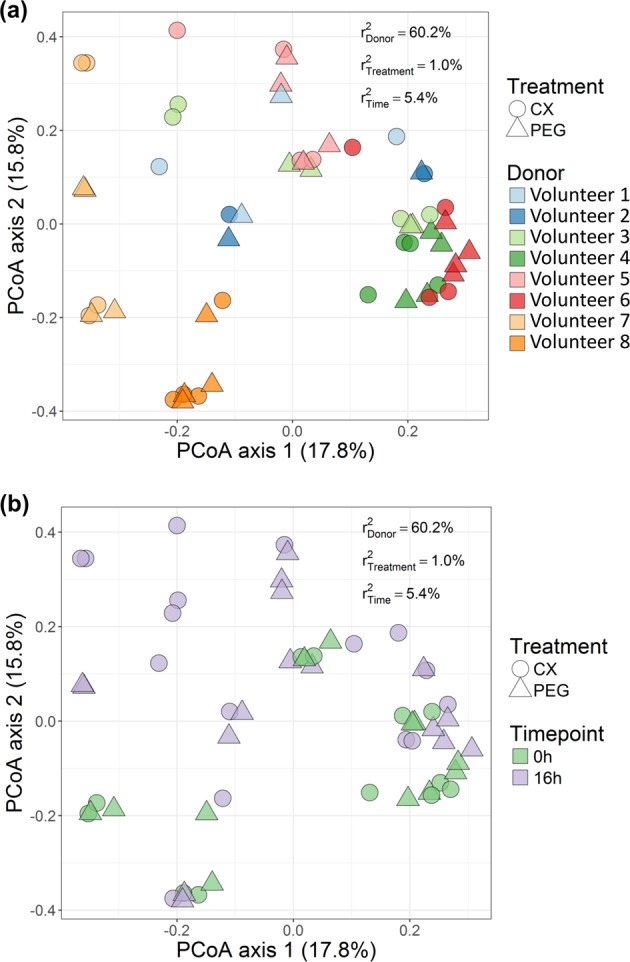
Interindividual differences drive bacterial community composition. Principal coordinate analysis (PCoA) revealed that **a** donor significantly contributed to variations among individual microbiomes, but not time **b**. The variance explained by experimental factors (*P* < 0.01) was calculated using PERMANOVA (top right) and confirmed differences in community structure between volunteers. *n* = 6 at 0 h, *n* = 8 at 16 h.

**Fig. 3:**
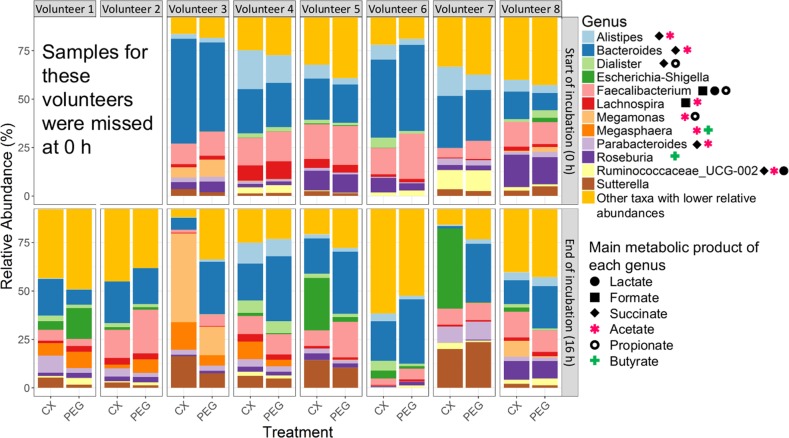
Relative abundances of bacterial genera occurring on each volunteer when celecoxib (CX) or a control treatment (PEG) were supplied. Bar plots include the metabolically active taxa with the highest relative abundances proportional to the entire microbiome, at the beginning (0 h, upper panel) and at the end of the experiment (16 h, lower panel). Data from volunteers 1 and 2 was not collected at the start of the experiment. Metabolic products of the taxa in the plots are included in the lower panel of the right.

Total DNA may also include that of dead bacterial cells, so we analysed community composition based on cDNA, as a proxy for the metabolically active bacterial community. Quantitative RT-PCR revealed that total 16S rRNA gene copy numbers from metabolically active bacteria were not significantly different between treatments, across donors (Supplementary Table 2). In contrast, community composition and structure tended to be influenced, as CX was associated with reduced but not significantly different Inverse Simpson alpha diversity index at the end of the incubation (Fig. 1, Table 2a, b). Permutational multivariate analysis of variance (PERMANOVA) confirmed that donor, but not treatment, significantly contributed to the differences in the mean relative abundances of bacterial genera (*P* < 0.001, Fig. 2, Table 2b). We observed that CX decreased alpha diversity when assessed individually (Supplementary Table 3, Fig. 3). Although interindividual variability was significant, the metabolically active taxa with the highest relative abundances proportional to the entire microbiome of control incubations were: *Bacteroides* sp., *Faecalibacterium* sp., Ruminococcaceae UGC002, and *Megasphaera* sp. In contrast, *Alistipes* sp., *Dialister* sp., *Escherichia*-*Shigella* sp., *Lachnospira* sp., *Megamonas* sp., *Parabacteroides* sp., *Roseburia* sp., and *Sutterella* sp. were highly abundant when CX was supplied (Fig. 3). Additionally, *Anaerostipes* sp., *Coprococcus* 2, Erysipelotrichaceae UCG 003, *Flavonifractor* sp., *Parabacteroides* sp., *Paraprevotella* sp., *Parasutterella* sp., *Phascolarctobacterium* sp., *Ruminoclostridium* 5 sp., *Ruminiclostridium* 6 sp., Ruminococcaceae UCG 010, *Ruminococcus* 2 sp., *Veillonella* sp., and *Victivallis* sp., were significantly different as a result of the donor*treatment*time point interaction (Supplementary 4). From all the community metrics, mean richness was the only one showing a trend for decrease when CX was supplied (*P* = 0.07, Table 2b) but our findings suggest that the individual microbiome is highly variable and is resistant to external stressors.

**Table 2:** Impact of donor and supplementation of celecoxib (CX) or vehicle (PEG) on bacterial community metrics at (A) the beginning of the incubation (0 h, *n* = 6) and (B) once the incubation was finalized (16 h, *n* = 8).

Metric	Index	Treatment		SEM	*P* value of each effect		
		CX	PEG		Treatment	Donor	Treatment*Donor interaction
(A)							
Evenness	Pielou	0.61	0.60	0.0009	0.83	0.0001	0.23
Alpha diversity	Shannon (*H*’)	2.71	2.72	0.03	0.81	<0.0001	0.13
	Simpson	0.85	0.85	0.007	0.43	<0.0001	0.04
	Inverse Simpson (*D*_2_)	8.00	8.48	0.53	0.54	<0.0001	0.76
	Fisher’s diversity	13.41	13.16	0.25	0.49	<0.0001	0.34
Richness	Total richness (*S*)	92.50	95.83	3.83	0.55	0.002	0.77
Abundance	Chao	144.12	128.55	9.54	0.27	0.01	0.59
(B)							
Evenness	Pielou	0.55	0.59	0.01	0.06	0.003	0.38
Alpha diversity	Shannon (*H*’)	2.54	2.70	0.06	0.08	0.001	0.33
	Simpson	0.84	0.86	0.009	0.14	0.009	0.10
	Inverse Simpson (*D*_2_)	7.59	8.13	0.63	0.55	0.02	0.23
	Fisher’s diversity	13.28	13.17	0.25	0.49	<0.0001	0.34
Richness	Total richness (S)	92.56	97.12	2.78	0.88	0.005	0.07
Abundance	Chao	94.01	114.65	5.34	0.01	0.008	0.89

### Short-term exposure to CX increases number of community metabolic networks

**Fig. 4:**
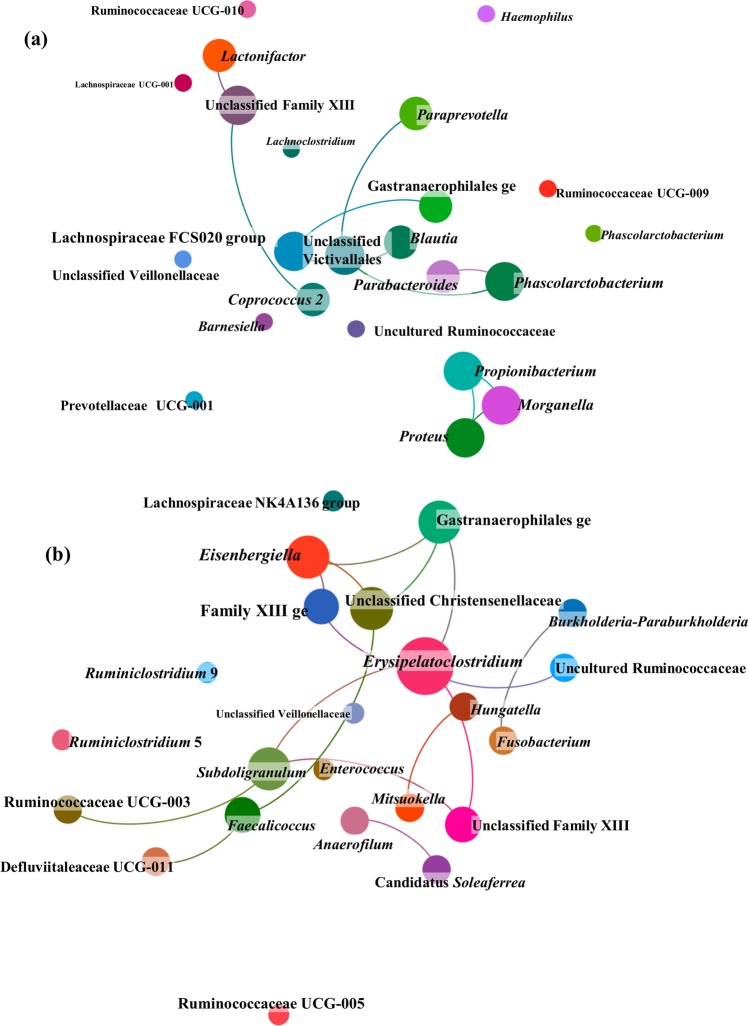
Bacterial network of communities exposed to short-term administration of celecoxib (CX) or its vehicle (control treatment, PEG). This representation uncovers relationships between clusters of genera across donors. **a** The faecal microbiota exposed only to PEG displayed a lower number of links, while the networks among taxa were enriched when CX was provided **b**. Nodes representing bacterial genera with similar abundances tended to cluster closely. Node diameter indicates the relative abundance of each genus. Different colours have been assigned to each node representing a bacterial genus. Neighbourhood selection method (MB) was the graphical model inference procedure selected. The associations described may be considered ‘core’ functional relationships preserved across donors.

Association networks were constructed to highlight potential shifts among bacterial relative abundances after short-term exposure to CX or its vehicle. The number of links in the network was lower when the vehicle PEG alone was provided (Fig. 4a). The central position of *Phascolarctobacterium*, *Parabacteroides*, *Coprococcus* 2 and Unclassified Victivallales and the linkages among these genera suggest concomitant changes in their relative abundances in the presence of PEG. The central position of the acetate-producing *Erysipelatoclostridium* in the CX network (Fig. 4b) indicated that its relative abundance was associated with *Subdoligranulum*, a butyrate and lactate-producing genus, in a donor-dependent manner (*P* < 0.05, Supplementary Table 4).

**Fig. 5:**
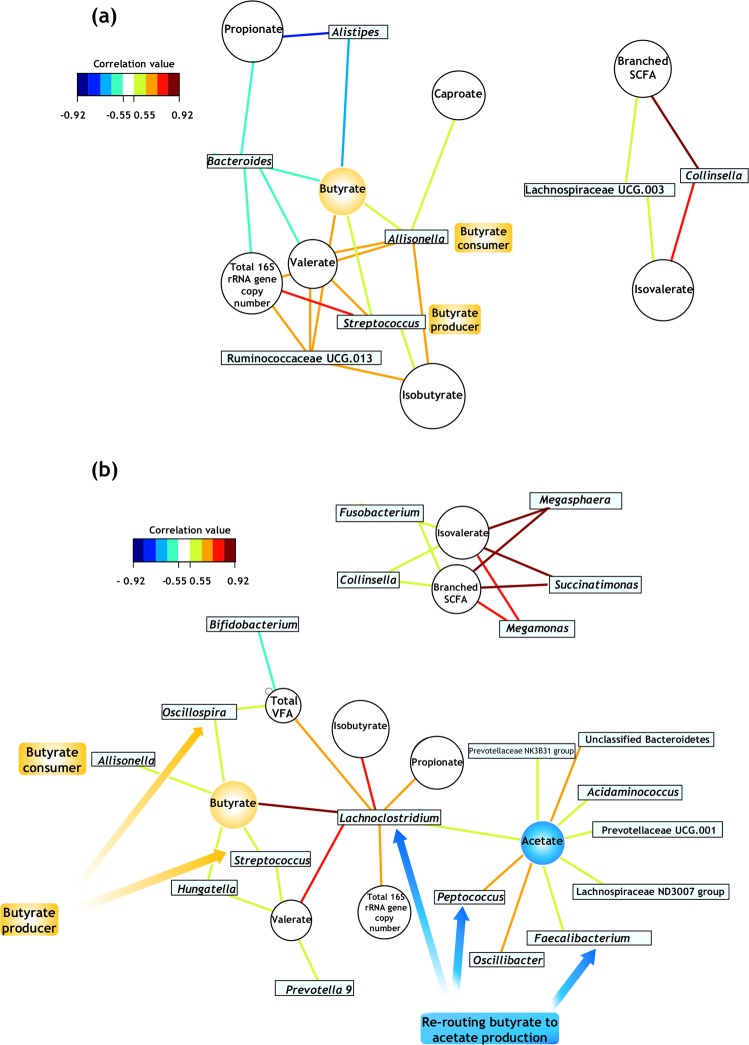
Community metabolic activity indicates that networks of bacterial interactions were few when the vehicle alone (PEG) was supplied **a** and increased following short-term supplementation of celecoxib **b**. These bipartite networks are based on the regularized canonical correlations between relative bacterial abundances and relative concentrations of the main short-chain fatty acids. Interactions have been filtered for an absolute correlation above 0.5 and are coloured following the key shown. Significant interactions are indicated by shorter lines, and genera with similar abundances within treatment tended to cluster closely.

Networks connecting bacterial load, metabolites and relative abundances were assembled to monitor functional alterations associated with changes in genera abundances. Activity-based relative abundance of *Bacteroides* was positively associated with butyrate, propionate and valerate levels and total 16S rRNA gene copy number in presence of PEG (Fig. 5a, Supplementary Table 4). These results confirmed that *Bacteroides* was one of the most abundant active genera, as first suggested in Fig. 3. Higher number of links in comparison with PEG were detected after 16 h of exposure to CX (Fig. 5). Indeed, one network linking acetate with the relative abundance of diverse taxa was only present when CX was supplied and contained the largest number of active genera participating. Relative abundance of *Faecalibacterium* decreased over time with CX supplementation, corresponding with the decline in butyrate levels (Fig. 3). However, this genus tended to be positively associated with acetate production (Fig. 5b). Relative abundance of active *Lachnoclostridium* was significantly associated with butyrate in the CX treatment (Fig. 5b). Here, *Oscillospira*, *Allisonella*, *Hungatella* and *Streptococcus* were linked because they possibly participated in butyrate production as well. *Megamonas* and *Megasphaera* were other genera with high relative abundances positively associated with branched-chain SCFA and isovalerate. The associations between the latter metabolites and *Colinsella* remained unchanged upon CX exposure, suggesting that this community function is either independent from drug supplementation or directly associated with PEG addition (Fig. 5a, b). Similarly, associations between *Allisonella* (butyrate consumer), *Streptococcus* (butyrate producer) and butyrate were common for both treatments. Results from the network analysis indicate that shifts in the community metabolic activity upon short-term supplementation of CX potentially impacted butyrate production.

### Inter-individual differences play a role in reduction of in vitro inflammation when CX is presented to the microbiome

Understanding the microbial interactions with the host epithelium is essential to assess whether the anti-inflammatory efficacy of CX remains unaltered following exposure to the simulated microbiome. The THP-1 cell line (macrophage-like) is known to be a suitable, simplified, and reliable model to study macrophage functions^28^ and inflammatory responses to external stimuli in the surrounding environment, such as drugs.^29,30^ We initially performed a mechanistic study to examine the inflammatory response mediated by CX using these cells. We further verified the microbiome impact using a multicellular model simulating the enterohepatic ecosystem, including enterocyte-like and goblet-like cell lines, plus THP-1 cells. This set-up enabled closer communication between cell lines,^31^ as would occur when macrophages infiltrate the underlying intestinal mucosa.^32^

We considered IL-8 and TEER appropriate indicative markers of intestinal inflammation, because they have been described as hallmark for inflammatory response and epithelial barrier function, both in vitro and in vivo.^33^ In addition to IL-8, another cytokine involved in intestinal inflammation (CXCL-16)^34^ was quantified.

**Fig. 6:**
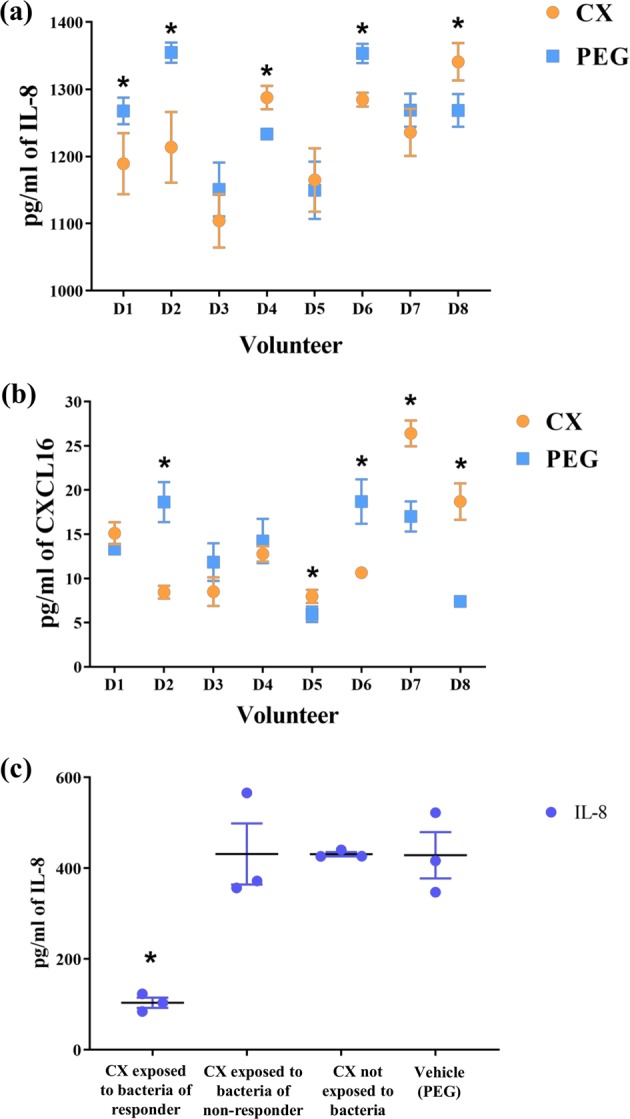
Concentration of pro-inflammatory cytokines was decreased in a donor-dependent manner when celecoxib was supplied. Volunteers are numbered consecutively as D1–D8. **a** IL-8 was significantly lower in the simplified inflammation model subjected to the bacteria-exposed celecoxib of three of the volunteers screened (D1, D2 and D6). Three additional volunteers (D3, D5 and D7) showed a trend for decreased IL-8, but it was not significantly lower than the control treatment. **b** CXCL16 was significantly lower in the simplified inflammation model subjected to the bacteria-exposed celecoxib from only two volunteers (D2 and D6) and tended to be decreased in other two (D3 and D4). **c** The two volunteers with the most extreme metabolic responses in butyrate production were selected to be screened in a multicompartment cell model resembling the enterohepatic system. Volunteer 6 (D6) had the most significant decrease in butyrate levels and was considered “responder” to the CX treatment. Volunteer 8 (D8) did not show any significant difference in butyrate between the control and the treatment after 16 h of incubation and it was considered as “non-responder”. IL-8 concentration was significantly lower in the model subjected to the supernatant of D6 containing bacteria-exposed celecoxib (“responder volunteer”). The opposite effect was observed in D8 (“non-responder volunteer”), suggesting that interindividual differences in the functionality of the microbiome may play a role in the response to anti-inflammatory drugs. *N* = 8, “*” indicates *P* < 0.05 between volunteers. Error bar indicates SEM.

Direct contact of macrophage-like cells with filter-sterilized supernatants from the CX-exposed microbiota resulted in a variable outcome in IL-8 and CXCL-16 concentrations. Compared with the PEG controls, a decrease in IL-8 and CXCL-16 was only observed in four out of eight individuals (Fig. 6a, b). Only two volunteers (D2 and D6) showed concomitant decrease of IL-8 and CXCL-16, while one person showed increased concentrations of both chemokines following supplementation with CX (D8) (Supplementary Table 5). These results suggest that successfully decreased inflammation potentially occurred with lowered recruitment and infiltration of leukocytes in the epithelial cells.

High IL-8 concentrations were found when non-bacterially exposed CX was applied in the enterohepatic model (Fig. 6c). In contrast, the lowest concentration of IL-8 and thus the most significant decrease in inflammatory response was found with the CX-exposed supernatant of donor 6 (*P* < 0.05, Table 3), which showed decreased butyrate concentrations and was considered “responder” to CX. CX that was not in contact with bacteria promoted the highest decrease in transepithelial electrical resistance (TEER) (*P* < 0.05, Table 3), in comparison with supernatants from CX-exposed bacteria. This outcome indicates less damage to epithelial barrier function and decreased inflammatory response when gut microbiota was exposed to CX.

**Table 3:** Epithelial integrity metrics of a multicompartment cell model simulating the enterohepatic metabolism.

Treatment	Time point	TEER	SEM	*P* value of each effect		
				Treatment	Time	Treatment*Time interaction
CX alone (100 mg/mL)	0 h 16 h	385.00 271.67^a^	34.61	0.007	0.01	0.40
PEG	0 h 16 h	330.67 338.67^a^	34.61	0.007	0.01	0.40
Donor 6 (responder) + CX	0 h 16 h	470.00 425.33^b^	34.61	0.007	0.01	0.40
Donor 8 (non-responder) + CX	0 h 16 h	398.33 311.33^a^	34.61	0.007	0.01	0.40
Control medium without LPS	0 h 16 h	421.33 337.33^a^	34.61	0.007	0.01	0.40
Control with LPS	0 h 16 h	428.67 433.00^a^	34.61	0.007	0.01	0.40

This cell model was subjected to supernatants of microbiome incubated with either CX or the carrier (PEG) for 16 h. Cell models were exposed to supernatants for an equal length of time, to simulate the transit time in the proximal colon. Inflammation was initiated with LPS and TEER was measured at 0 h (prior to LPS exposure) and at 16 h (after 16 h of incubation with LPS). Different superscripts indicate significantly different means across treatments, within a particular time point. *N* = 3.

## Discussion

The lipophilic characteristics of CX favour metabolic elimination,^35^ and exploring only the pathways for CX distribution in the host is insufficient for assessing drug bioavailability. In vitro models have been applied to simulate drug-bug associations in the human gut,^36,37^ but the impact of NSAIDs on the metabolic activities of the microbiome has been overlooked. Previous studies reported the influence of NSAIDs on the gut microbiome,^38^ yet no changes in bacterial richness, microbiome composition or beta diversity were found when CX was supplied.^20^ These results screened community structure using DNA-based amplicon sequencing, which may also include DNA of dead bacterial cells. As such approach may deliver a partial overview, we used cDNA as an indicator of the metabolically active bacterial population and we performed community composition analyses based on information from transcribed RNA.^39^ In this way, we intended to highlight the main bacterial genera in the RNA pool whose metabolic activities were impacted by CX. We confirmed that mean activity-based cell load, composition, diversity and richness were not significantly different among donors, and the only alterations were on the bacterial metabolic activities. PERMANOVA analysis suggested that donor was the main driver of the interindividual dissimilarities in the most abundant genera between treatments.

The similar total active bacterial load observed between treatments may indicate that CX mainly inhibited functional activities of groups of bacteria rather than those of single genera, ultimately impacting community fermentation.^40^ CX users have been reported to have enrichment of Enterobacteriaceae and Acidaminococcaceae.^38^ Here, we validated that the relative abundance of transcriptionally active *Escherichia*-*Shigella* (Enterobacteriaceae) was increased with CX supplementation, whereas the relative abundance of active *Acidaminococcus* (Acidaminococcaceae) was positively associated with acetate in the same treatment. Coriobacteriaceae (*Colinsella*) has been proposed to participate in xenobiotic metabolism by decreasing levels of serine and glycine,^40^ which can be both fermented to acetate in the gut.^41^ We observed that the relative abundance of this genus was positively associated with isovalerate levels when CX was added. As indicated on its corresponding KEGG module, *Colinsella aerofaciens* has the capacity of using isovalerate for synthesizing leucin and serine,^42^ confirming its potential xenobiotic role in our study. These results highlight the applicability of our in vitro model for examining drug-microbiome interactions. The capacity of *Alistipes* for producing succinate^43^ may explain its association with propionate in the PEG treatment. *Dialister* was another succinate producer^44^ present at high relative abundance, which does not seem to participate in the propionate network when PEG was supplied. This suggest that high relative abundance of transcriptionally active taxa may be result of increased maintenance metabolism of that single taxa and not due to cross-feeding interactions.

Beyond its tumour inhibitory properties,^45^ butyrate ameliorates mucosal inflammation and oxidative status, reinforces the epithelial defence barrier, and modulates visceral sensitivity and intestinal motility.^46^ Thus, the inflammation-butyrate interplay is not only a direct effect of altered SCFAs concentrations, but also associated with changes in microbiome functionality. The significantly lower butyrate concentration post-CX delivery may have several implications. Indeed, the protecting properties of CX towards CRC^47,48^ may be partially derived from lowering bacteria-derived butyrate. Nutrients can be utilized in their final form by both host and gut microbes, leading to competition for resources.^49^ In fact, blocking fatty acid supply and host recapture has been proposed as strategy for influencing cancer cell bioenergetics,^50,51^ but few studies have addressed the potential role of acetate in cancer.^52^ Acetyl-CoA is the main carbon source in mammalian cells, and the ligation of acetate and CoA by acetyl-CoA synthetase (ACSS) contributes to the supply of this metabolite.^52^ Cancer cells use acetyl Co-A during aerobic metabolism regardless of stress conditions.^52^ Butyrate is also metabolized to acetyl-CoA,^45^ inhibiting tumour growth via silencing acetyl-CoA synthetases^50^ or potentially through acetate uptake. As acetyl-CoA is a bacterial substrate for fatty acid production,^53^ our network analysis suggests that acetate was used for bacterial metabolic functions when CX was supplied. Thus, CX may favour bacterial competition for acetyl Co-A, generating potential host protective effects. Indeed, microbiota-derived acetate has been associated with inhibition of cancer-associated deacetylation.^54^ Increasing acetate concentration shifts SCFA ratios,^55^ decreasing butyrate and explaining why the acetate:propionate:butyrate ratios tended to change when CX was supplied. *Faecalibacterium* sp. is a well-known butyrate producer and acetate consumer^55^ and its presence in the acetate network may indicate that the relative abundance of this genus increased when CX was provided and acetate levels increased.

Synthesis of reduced products including H_2_, succinate, and butyrate creates redox balance during colonic fermentation, whereas formation of oxidized products, such as acetate, is associated with bacterial ATP production.^53^ Fermentative activities of *Lachnoclostridium* yield ethanol, acetate, CO_2_ and H_2_,^56,57^ explaining the numerous links with other genera in the CX network. These potential cross-feeding interactions,^58,59^ may support its positive association with the total 16S rRNA gene copy number. Acetate is a co-substrate for butyrate production,^60^ but butyrate production may be reduced when NADH is used for H_2_ formation during glycolysis. Thus, NADH is not available to convert acetoacetyl-CoA to butyryl-CoA and butyrate concentrations decrease.^53^ Acetate-producing *Lachnoclostridium* and *Oscillospira* possess transporters involved in uptake of oligosaccharides and glycerol-P,^58^ explaining their potential involvement in the shift from butyrate to acetate production when CX was supplied. Additionally, Peptococcaceae uses the acetyl-CoA pathway in a reverse direction to oxidize butyrate.^61^ Acetyl Co-A is consequently routed into acetate formation,^60^ which is energetically advantageous for bacterial use.^53^ In fact, butyrate producers may shift metabolic pathways towards acetate under stress conditions.^62^ Previous research showed that other acetate producers such as Bacteroidetes and Prevotellaceae positively correlated to butyrate-producing communities using the acetyl CoA pathway for butyrate production,^61^ supporting the potential shift in butyrate towards acetate observed in our study.^63^

Although the role of SCFA on chronic inflammatory diseases has been documented, most of the studies lack natural commensal microbiota cross-talking with host cells.^45,64^ Interindividual differences in microbiome significantly impact drug responses,^16^ and thus it is paramount elucidating whether their microbiome fermentation products and NSAIDs work together for resolving inflammation. CX was responsible for only 5% of the variance in bacterial community composition among the eight donors, but the effect of CX-exposed bacteria on the proinflammatory IL-8 was sufficient to be detected in a donor-dependent manner. Moreover, samples derived from 50% of the donors showed increased CXCL-16, which effectively reduces pro-inflammatory macrophages in the liver^65^ and is a marker of favourable prognosis in CRC.^66^ Butyrate specifically suppresses proinflammatory effectors in lamina propria macrophages.^67^ The results observed in the cell model suggest that the interaction between CX and microbiome to resolve inflammation may be not exclusively related to community composition or bacterial abundance, and microbiome functionality must be considered. Thus, when either the abundance or activity of butyrate producers is lowered in chronic inflammation,^68,69^ CX may compensate for the anti-inflammatory effect of butyrate and support the resolution of the inflammatory process. While butyrate seemed to be reduced with short-term treatment, Acetate:Propionate:Butyrate ratio and relative levels of acetate (of the total SCFA) may be other bacterial metabolism variables relevant for assessing successful reduction on inflammation following NSAIDs administration. Hence, using multicompartment cell models for future studies assessing the crosstalk of the microbiota and colon primary cells or organoids, in the context of chronic inflammatory diseases, are imperative.

As indicated in our study, gut microbiome activities and its interplay with the host can be modulated through CX. Certainly, individual microbiome may need to be considered before supplementing CX as a colon-targeted drug in the context of chronic inflammation and CRC. Divergence in the molecular approaches (i.e. next generation sequencing, qPCR) used for investigating inflammation-associated microbiome signatures contributes to the lack of consensus on the composition of microbiota in patients with chronic inflammatory diseases. Our in vitro model retained interindividual variability, allowing for mechanistic research on drug-microbiota-host interplay, fundamental for developing individualized therapeutic strategies. Focusing on functionality rather than taxonomic diversity may be the next frontier for developing predictive markers of inflammation-induced dysbiosis and NSAID efficacy.

## Methods

### Faecal incubations simulating the proximal colon environment

Batch incubations of faecal samples were conducted using phosphate-buffered nutritional medium (pH 5.9, 0.1 M) containing (per litre): 12.25 g of KH_2_PO_4_ (Carl Roth GmbH, Karlsruhe, Germany), 1.78 g of Na_2_HPO_4_ (Carl Roth GmbH, Karlsruhe, Germany), 3 g of yeast extract (Oxoid Ltd, Basingstoke, Hampshire, England), 1 g of proteose peptone (Oxoid Ltd, Basingstoke, Hampshire, England), 0.25 g of gum Arabic from acacia tree (Sigma Aldrich Co., St-Louis, USA), 0.5 g of apple pectin (Sigma Aldrich Co., St-Louis, USA), 0.25 g of xylan (Carl Roth GmbH, Karlsruhe, Germany), 1 g of potato starch (Prodigest, Zwijnaarde, Belgium), and 4 g of mucin from porcine stomach Type II (Sigma Aldrich Co., St-Louis, USA).

Faecal material was obtained from eight healthy, non-obese, non-smoking volunteers (4M and 4F, 30 ± 2.8 yo), following an omnivorous diet and without antibiotic exposure for the prior 6 months. Stool samples were collected in a closed recipient under microaerophilic conditions (AnaeroGen, Oxoid, Hampshire, UK) and homogenized 1:5 (w/v) in anaerobic potassium phosphate buffer (0.1 M at pH 7.0) containing 1 g/L of sodium thioglycolate as reducing agent, and centrifuged during 2 min at 500 × *g* to obtain faecal slurries of 20% (w/v). CX (Janssen Pharmaceutica, Beerse, Belgium) was dissolved in 70:30 (v/v) PEG_400_:H_2_O as vehicle, at a stock concentration of 100 mg/mL. The administered dose was established based on data from the manufacturer and intended for a colon-targeted formulation. For each donor, triplicate incubations of 10% faecal slurry, 100 mg/mL of CX or 70:30 (v/v) PEG_400_ (Acros, Geel, Belgium):H_2_O, and nutritional medium (final volume = 175 mL) were performed for 16 h at 37 °C in a shaking incubator at 120 rpm (New Brunswick Scientific InnOva 4080 Incubator Shaker, Wezembeek-Oppem, Belgium), under anaerobic conditions (90%–10% N_2_/CO_2_). Once the incubation was completed, samples were collected, flash-frozen using liquid nitrogen and preserved at −80 °C for further analysis. Samples from volunteers 1 and 2 were missed at the 0 h collection point.

### Surveying community functionality and composition

#### SCFAs production

SCFA were used as benchmarks of community activity. Extraction from samples was performed with diethyl ether, after the addition of 2-methyl hexanoic acid as an internal standard. Extracts were analysed using a GC-2014 gas chromatograph (Shimadzu, ‘s-Hertogenbosch, the Netherlands), equipped with a capillary fatty acid-free EC-1000 Econo-Cap column (dimensions: 25–0.53 mm, film thickness 1.2 mM; Alltech, Laarne, Belgium), a flame ionization detector and a split injector. The injection volume was 1 mL and the temperature profile was set from 110 to 160 °C, with a temperature increase of 6 °C/min. The carrier gas was nitrogen and the temperature of the injector and detector were 100 and 220 °C, respectively. Differences in SCFA concentrations among treatments were compared using a repeated measures mixed model,^70^ with the lsmeans adjustment and Bonferroni correction for multiple comparisons (SAS, 2012). Statistical significance was assumed at *P* < 0.05.

#### Quantification of total metabolically active bacterial community

Samples collected from the faecal incubations at 0 h (start) and after 16 h (end of experiment) were thawed on ice, centrifuged for 5 min at 4 °C and 5000 × *g*, and disrupted two times for 25 s at 1400 rpm using a homogenizer instrument (Power Lyser 24, MO BIO Laboratories, Carlsbad, USA), with a 5-s interval, where the samples were placed on ice. RNA was further extracted using the Nucleospin RNA plus kit (Macherey Nagel, Düren, Germany), following the manufacturer’s instructions. Reverse transcription was completed using the Reverse Transcriptase Core kit (Eurogentec, Seraing, Belgium).

For the quantification of total bacterial 16S rRNA gene copy numbers, a standard curve was constructed using serial dilutions of plasmid DNA from a clone identified as *Bifidobacterium breve* LMG 33264. Briefly, universal bacterial primers 27F and 1492R were used to amplify the full-length 16S rRNA gene from the plasmid DNA of a *Bifidobacterium breve* clone. The resultant PCR product was purified using the InnuPREP PCRpure Kit (AJ innuscreen, Berlin, Germany), cloned in *E. coli* with the TA Cloning Kit (Life Technologies, Carlsbad, VS) and plasmid extracted with the PureYield Plasmid Miniprep System (Promega, Leiden, The Netherlands). The mass concentration of the plasmid was measured using an ND-1000 spectrophotometer (NanoDrop Technologies, Wilmington, DE), converted to the molecule concentration^71^ and copy numbers of total bacteria in 50 ng of cDNA and per mL of faecal incubation were determined by relating the threshold cycle (*C*_T_) values to the standard curves based on the following equation: *Y* = −3.193 × log*X* + 35.003 (*Y*, *C*_T_ value; *X*, copy number of 16S rRNA gene) (*r*^2^ = 0.996).^71^

Quantitative reverse transcription-PCR (qRT-PCR) analysis of the metabolically active community was achieved with the StepOnePlus Real-Time PCR system (Applied Biosystems, Foster City, CA, USA). Amplification reactions were in: 10 × Taq buffer with KCl, 25 mM of MgCl_2_, 10 mM of dNTP mix, 10 µM of each forward and reverse primers, 20 mg/mL of BSA (Thermo Scientific, Waltham, USA), 1.25 U of Taq Polymerase, 50 ng of cDNA and 0.1 × SYBR Green I ([Invitrogen, Carlsbad, USA] provided at 10 000×, stock solutions of 20× were prepared in DMSO). qRT-PCR conditions included 10 min at 95 °C, followed by 40 cycles of 15 s at 95 °C and 30 s at 60 °C. The efficiencies (*E*) of RT-PCR were calculated from the given slopes in StepOnePlus software using the following equation: *E* = [10(^−1/slope^) − 1] × 100%. Data generated from reactions with efficiencies between 90% and 110% were used for further analysis.^71^ Differences in copy numbers among treatments were compared using a repeated measures mixed model,^70^ with the lsmeans adjustment and Bonferroni correction for multiple comparisons (SAS, 2012). Statistical significance was assumed at *P* < 0.05.

### Community composition and dynamics

The V5–V6 hypervariable region of the 16S rRNA gene from the cDNA was amplified using primers 341F and 785R. High-throughput amplicon sequencing was performed with the Illumina MiSeq platform according to the manufacturer’s guidelines at LGC Genomics GmbH (Berlin, Germany). Library preparation and purification as well as bioinformatic processing are described in the supplemental material.

Data was imported into R using *phyloseq*^72^ and taxon abundances were rescaled by calculating the taxon proportions and multiplying them by the minimum sample size (*n* = 2201) present in the data set.^73^ Inverse Simpson was the metric used for assessing alpha diversity and Pielou’s index was used as indicator of community evenness.^74^ Differences in alpha diversity and evenness measures were compared among treatments using a repeated measures mixed model in SAS (version 9.4, SAS Institute, Cary, USA). Beta diversity based on Chao and Bray-Curtis indices was used to examine dissimilarity and determine the impact of treatment and time on microbial community structure.^75^ PCoA was employed to visualize the differences among samples, using the vegan package in R.^76^ Stratified PERMANOVA with 999 permutations was conducted to indicate the significance of each covariate (time or treatment) on the microbial community.

ANOVA was applied to reveal whether the distribution of the genera was different between treatments over time.^76^ Because of the over-dispersion in the OTU data, a zero-inflated negative binomial distribution count model was used to assess the effect of donor, time and treatment and the interactions between donor*time*treatment on each individual genus. The model was selected based on the Akaike Information Criterion (AIC). Differences among library size sample were accounted for with the offset option in proc GLIMMIX in SAS.^75^

Sparse inverse covariance selection was employed to infer an association network within treatments, using the package SpiecEasi (v0.1.2)^77^ in R (v3.5.1). Neighbourhood selection method (S-E(MB) was used to construct a network of bacterial genera displaying core networks with either functional or phylogenetic similarities. The calculated adjacency matrix was examined with the software Gephi 0.9.2,^78^ using the Fruchterman-Reingold algorithm for visualizing significant correlations (*R* ≥ 0.7). This method computes a layout in which the length of the connections indicates the absolute value of the correlation. The nodes representing genera whose relative bacterial abundances have several significant correlations among them were placed closer to each other in the network.^79^

Bipartite networks highlighted functional associations among bacterial genera and metabolites,^75^ using a pair-wise similarity matrix obtained from a regularized canonical correlation analysis.^80^ Values of the similarity matrix were computed as the correlation between relative abundances of bacterial genera and metabolic variables, projected onto the space spanned by the first three components retained in the analysis (*r* ≥ 0.75). Genera in the plot were close to correlated variables in the treatment where they were more abundant.^75^

### Assessing the anti-inflammatory potential of bacteria-exposed CX

#### Simplified inflammation model

The THP-1 cell line (macrophage-like) is known to be a suitable, simplified, and reliable model to study macrophage functions.^28^ The human monocytic leukaemia cell line THP‐1 (THP-1, ECACC 88081201, Public Health England, UK) was grown in Roswell Park Memorial Institute (RPMI) 1640 culture medium (Thermo Fisher Scientific, Waltham, USA) supplemented with 10% heat-inactivated foetal bovine serum (iFBS) (Greiner Bio-One, Wemmel, Belgium) and 1% antibiotic-antimycotic solution (10,000 units/mL of penicillin, 10,000 µg/mL of streptomycin, and 25 µg/mL of Amphotericin B) (Thermo Fisher Scientific, Waltham, USA), at 37 °C in 10% CO_2_ in a humidified incubator. THP‐1 cells were sub‐cultured twice a week and used between passages 20 and 30.

Macrophage-like phenotype was obtained by treating cells with 25 ng/mL phorbol 12‐myristate 13‐acetate (PMA, Sigma Aldrich, St-Louis, USA) for 48 h in 24‐well culture plates (Corning, Sigma Aldrich, St-Louis, USA) at a density of 1 × 10^5^ cells/cm^2^. Differentiated, plastic‐adherent cells were washed twice with culture medium (supplemented RPMI 1640 without PMA) and incubated for 24 h. The cell morphology was analysed by a Zeiss Primovert inverted microscope (Oberkochen, Germany). Medium was removed and replaced by supernatant from the eight incubations, passed through a sterile 0.2 µm filter and diluted five times with RPMI 1640 without supplementation. CX solution at 100 mg/mL dissolved in 70:30 (v/v) PEG:H_2_O or 70:30 (v/v) PEG:H_2_O alone were diluted 1:5 with medium and added as controls of non-bacteria exposed CX and vehicle. This dilution step was performed to simulate low solubility and the small aqueous volume in the colon.^18^ LPS (100 ng/mL) dissolved in RPMI 1640 was added to the cells to simulate systemic inflammation, as in CRC. The control used in all measurements was PMA-differentiated THP‐1 macrophages exposed to the culture medium.

#### Development of an in vitro model of the enterohepatic system

A novel model including intestinal epithelial (Caco-2, ECACC 86010202), goblet (HT29-MTX-E12 ECACC 12040401, Public Health England, UK), hepatic (HepG2 ATCC HB-8065, Molsheim, France), and immune-like cells (THP-1, ECACC 88081201, Public Health England, UK) was developed to study host-drug-microbiome interactions.^31,81^ Cell layers were grown in a double-chamber insert (Corning, Sigma Aldrich, St-Louis, USA), and maintained for 23 days. Briefly, 100 µL of a THP-1 cell suspension (10^6^ cells/mL) in 20 mg/mL collagen from rat tail type I (Sigma Aldrich, St. Louis, USA) were spread in the basolateral surface of inverted inserts. After 45 min of incubation at 37 °C and 10% CO_2_, a 9:1 cell suspension of Caco-2/HT29-MTX cells (density of 7.5 × 10^4^ cells/cm^2^) was added to the apical side of the insert in 1.5 mL of Dulbecco’s modified Eagle’s medium (DMEM) supplemented with 10% (v/v) of iFBS and 1% of antibiotic-antimycotic solution (v/v). The basolateral compartment of the double-chamber system was filled with 2 mL of supplemented RPMI 1640, and the medium was refreshed every 48 h. Inserts were transferred to six-well plates with pre-seeded HepG2 cells at day 19 post-seeding, when the cell culture media in the basal compartment was replaced by RPMI 1640, and the co-culture was maintained 24 h prior to the assay. HepG2 cells were seeded at a density of 10^5^ cells/cm^2^ and maintained 5 days in supplemented minimal essential medium (MEM) supplemented with 10% (v/v) of iFBS and 1% (v/v) of antibiotic-antimycotic solution. Five days prior the assay all the cell culture media was depleted of antibiotic/antimycotic solution. Cells were negative for *Mycoplasma* contamination. The cell morphology was analysed by a Zeiss Primovert inverted microscope (Oberkochen, Germany). Supernatants from volunteers (2F, 29 yo) showing extreme microbiota metabolism responses in the short-term incubations were used for validating whether CX effectively decreases inflammation even after exposure to the gut microbiota. Volunteer 6 displayed the most significant decrease in butyrate after 16 h of incubation and was identified as “responder” to CX supplementation. Volunteer 8 did not present any changes in fermentation products and was termed “non-responder”. Triplicate supernatants from each volunteer were passed through a sterile 0.2 µm filter, diluted five times with DMEM without supplementation, pH was adjusted to pH 7–7.2 with filter-sterilized NaOH 0.5 M (Carl Roth GmbH & Co), and placed in the apical compartment of the model. Results from the simplified inflammation model indicated that there were no significant differences between control and treatment among donors. Thus, we only included CX solution dissolved in 70:30 (v/v) PEG:H_2_O or 70:30 (v/v) PEG:H_2_O alone in the enterohepatic model. CX solution at 100 mg/mL and a 70:30 (v/v) PEG:H_2_O were diluted 1:5 with DMEM without supplementation, and added to the apical compartment, as controls of non-bacterially modified CX and vehicle. This dilution step was performed to simulate low solubility and the small aqueous volume in the colon.^18^ LPS (100 ng/mL) was added to the basal compartment to simulate systemic inflammation. Plates were incubated for 16 h at 37 °C, 10% CO_2_ and 95% humidity. TEER was evaluated with the Millicell ERS-2 Voltohmmeter at the beginning and at the end of the assay (Merck-Millipore, Overijse, Belgium). Differences in TEER among treatments were compared using a repeated measures mixed model in SAS,^70^ with the lsmeans adjustment and Bonferroni correction for multiple comparisons, with significance at *P* < 0.05.

#### Evaluating inflammatory response

Concentration of IL-8 and CXCL16 were measured in the simplified inflammation model with the Human IL-8 (CXCL-8) and Human CXCL16 Mini ABTS ELISA Development Tests (PeproTech, London, UK) and the ABTS ELISA Buffer Kit (PeproTech, London, UK), following the manufacturer’s instructions. Samples were not diluted, and determination was performed at 405 nm, with correction for 650 nm (Infinite 200 PRO, Tecan, Männedorf, Switzerland). IL-8 was additionally determined in the presystemic metabolism model, using samples from the basal compartment diluted 10 times with PBS (ABTS ELISA Buffer Kit, PeproTech, London, UK), and measured as indicated above. Multiple pairwise comparisons were completed in GraphPad (GraphPad Prism 8, La Jolla, CA, USA) using one-way Anova and Tukey’s test. Statistical significance was assumed at *P* < 0.05.

### Reporting summary

Further information on research design is available in the Nature Research Reporting Summary linked to this article.

## Supplementary information

Reporting Summary

Supplementary material

## Data Availability

Raw 16S rRNA reads have been made available on the SRA under accession number ID PRJNA540406.

## Code availability

Original R scripts are available in GitHub (https://github.ugent.be/ehernand/CX_sequencing).
