# Supplementary material for: Short-term supplementation of celecoxib-shifted butyrate production on a simulated model of the gut microbial ecosystem and ameliorated in vitro inflammation
Source: NPJ Biofilms Microbiomes. 2020 Feb 19;6:9. doi: 10.1038/s41522-020-0119-0 (PMC7031363; doi:10.1038/s41522-020-0119-0)
Supplement: Supplementary file 2 — Supplementary material [file 41522_2020_119_MOESM2_ESM.pdf]

SUPPLEMENTARY MATERIAL

Short term supplementation of celecoxib shifted butyrate production on a simulated model of the microbial ecosystem and ameliorated *in vitro* inflammation

Emma Hernandez-Sanabria[1], Evelien Heiremans[1], Marta Calatayud Arroyo[1], Ruben Props[1], Laurent Leclercq[2], Jan Snoeys [2], and Tom Van de Wiele[1]

[1] Center for Microbial Ecology and Technology (CMET), Ghent University, Coupure Links 653, 9000 Ghent, Belgium. [2] Janssen Research & Development, A Division of Janssen Pharmaceutica NV, Turnhoutseweg 30, Beerse, Antwerp, 2340, Belgium.

Running title: Celecoxib shifts butyrate in an *in vitro* gut microbial ecosystem

Supplementary Table 1. Bacterial functionality metrics following short-term incubation (16 h) with either celecoxib or carrier (n = 8). CX, celecoxib; PEG, carrier. SEM, standard error of the mean.

Variable	Treatment (Mean ± SEM)		*P* value
	CX	PEG	
Acetate (mM)	11.51 ± 1.44	13.31 ± 1.50	0.39
Propionate (mM)	3.29 ± 0.41	3.86 ± 0.42	0.33
Butyrate (mM)	1.57 ± 0.33	3.20 ± 0.33	0.0007
Valerate (mM)	0.61 ± 0.15	0.78 ± 0.15	0.45
Isobutyrate (mM)	0.30 ± 0.07	0.34 ± 0.07	0.70
Isovalerate (mM)	2.70 ± 0.52	3.30 ± 0.54	0.42
Caproate (mM)	0.15 ± 0.04	0.12 ± 0.04	0.61
Isocaproate (mM)	0.001 ± 0.002	0.01 ± 0.002	0.009
Total SCFA (mM)	20.14 ± 2.37	24.91 ± 2.45	0.16
Total Branched SCFA (mM)	2.56 ± 0.49	3.25 ± 0.52	0.34
St:Br ratio	17.76 ± 4.48	12.71 ± 4.31	0.42
% of Acetate	60.87 ± 2.60	53.58 ± 2.77	0.06
% of Propionate	16.17 ± 0.65	15.75 ± 0.70	0.67
% of Butyrate	6.57 ± 0.64	12.99 ± 0.68	<0.0001
Acetate:Propionate:Butyrate ratio	61:16:7	54:16:13	-
pH	5.76 ± 0.04	5.75 ± 0.04	0.86
Total copy number of 16S rRNA gene	3.37E09 ± 3.33E08	4.35E09 ± 3.33E08	0.04

Supplementary Table 2. Total metabolically active bacterial population at the beginning (A) and at after 16h (B) of incubation with either celecoxib (CX) or carrier (PEG). Means presented were calculated from 3 replicate incubations run in triplicate per donor. n = 6 at 0 h, n = 8 at 16 h; NS, not significantly different; SEM, standard error of the mean.

	0 h				*16 h*			
Donor	Treatment		SEM	*P* value	Treatment		SEM	*P* value
	CX	PEG			CX	PEG		
D1	-	-	-	-	9.78E+09	2.11E+10		<0.001
D2	-	-	-	-	4.90E+09	4.72E+09		NS
D3	1.54E+08	4.34E+07	4.11E+07	NS	8.59E+08	2.86E+08		NS
D4	1.49E+09	1.20E+09	3.88E+08	NS	1.79E+09	1.71E+09	9.41E+08	NS
D5	3.11E+09	9.08E+09	2.54E+09	NS	3.00E+09	4.73E+09		NS
D6	5.70E+08	3.47E+09	4.25E+08	0.0007	2.49E+08	2.78E+08		NS
D7	2.32E+08	3.43E+08	7.87E+07	NS	1.08E+09	1.64E+09		NS
D8	6.53E+08	1.12E+09	1.36E+08	0.04	5.30E+09	3.21E+08		0.04

Supplementary Table 3. Alpha diversity using inverse Simpson index (D_2) after exposure to celecoxib (CX) or the carrier (PEG). n = 6 at 0 h, n = 8 at 16 h; NS, not significantly different.

Volunteer	Time point	Inverse Simpson (Mean)		SEM	*P* value of each effect		
		CX	PEG		Time	Treatment	Time*Treatment
D1	0h<br>16h	-<br>7.20	-<br>11.16	4.41	-	NS	-
D2	0h<br>16h	-<br>6.08	-<br>7.89	1.85	-	NS	-
D3	0h<br>16h	3.32<br>3.90	4.33<br>7.43	0.42	0.006	0.01	0.04
D4	0h<br>16h	6.93<br>12.80	9.13<br>7.14	0.60	0.04	0.03	0.003
D5	0h<br>16h	10.42<br>6.54	9.51<br>6.48	1.16	NS	0.04	NS
D6	0h<br>16h	5.41<br>6.81	4.16<br>7.50	0.58	NS	0.01	NS
D7	0h<br>16h	8.86<br>4.57	9.99<br>6.26	0.99	NS	0.01	NS
D8	0h<br>16h	13.11<br>12.85	13.76<br>11.20	1.79	NS	NS	NS

Supplementary Table 4. Impact of donor, treatment and time on bacterial relative abundances following short-term supplementation of either celecoxib or the carrier. CX, celecoxib. PEG, carrier. Tpt, time point effect; Trt, treatment effect. n = 6 at 0 h, n = 8 at 16 h; NS, not significantly different.

Taxa	Time point	CX		PEG		*P* value				
		Mean	SEM	Mean	SEM	Donor	Tpt	Trt	Trt*Tpt interaction	Donor*Trt*Tpt interaction
Allisonella	0h	0.0001	0.00003	0.0002	0.0001	0.02	0.07	NS	NS	NS
	16h	0.0002	0.0001	0.0003	0.0001					
Anaerostipes	0h	0.0015	0.0004	0.0015	0.0003	0.002	NS	NS	NS	0.001
	16h	0.0008	0.0002	0.0013	0.0002					
Coprococcus 2	0h	0.0016	0.0005	0.0010	0.0003	<0.0001	NS	NS	NS	0.02
	16h	0.0008	0.0003	0.0010	0.0002					
Erysipelotrichaceae UCG 003	0h	0.0017	0.0003	0.0014	0.0002	0.003	NS	NS	NS	<0.0001
	16h	0.0015	0.0003	0.001	0.0002					
Flavonifractor	0h	0.0003	0.0001	0.0002	0.0001	0.001	NS	NS	0.08	0.0004
	16h	0.0001	0.00003	0.0002	0.0001					
Fusobacterium	0h	0.0016	0.0005	0.0012	0.0004	<0.0001	NS	NS	NS	NS
	16h	0.0015	0.0005	0.0015	0.0004					
Lachnospiraceae FCS020 group	0h	0.0001	0.00004	0.0002	0.0001	0.01	NS	NS	NS	NS
	16h	0.0001	0.00002	0.0001	0.00003					
Lachnospiraceae UCG 001	0h	0.0012	0.0003	0.0011	0.0002	0.11	0.004	NS	NS	NS
	16h	0.0004	0.0002	0.0005	0.0001					
Marvinbryantia	0h	0.0001	0.00003	0.00004	0.00001	0.17	<0.0001	NS	NS	NS
	16h	0.000004	0.000001	0.00001	0.000002					

Taxa	Time point	CX		PEG		*P* value				
		Mean	SEM	Mean	SEM	Donor	Tpt	Trt	Trt*Tpt interaction	Donor*Trt*Tpt interaction
Parabacteroides	0h<br>16h	0.0095<br>0.0109	0.0017<br>0.0014	0.0075<br>0.0080	0.0014<br>0.0009	<0.0001	NS	NS	NS	<0.0001
Paraprevotella	0h<br>16h	0.0005<br>0.0003	0.0002<br>0.0001	0.0005<br>0.0004	0.0002<br>0.0001	0.0001	NS	NS	NS	0.02
Parasutterella	0h<br>16h	0.0013<br>0.0020	0.0003<br>0.0004	0.0013<br>0.0013	0.0003<br>0.0003	<0.0001	NS	NS	NS	0.02
Peptococcus	0h<br>16h	0.0003<br>0.0001	0.0001<br>0.00004	0.0001<br>0.0001	0.00004<br>0.00003	0.01	0.06	NS	NS	NS
Phascolarctobacterium	0h<br>16h	0.0035<br>0.0027	0.0008<br>0.0006	0.0028<br>0.0029	0.0006<br>0.0005	<0.0001	0.56	NS	NS	<0.0001
Ruminiclostridium 5	0h<br>16h	0.0006<br>0.0003	0.0001<br>0.0001	0.0005<br>0.0003	0.0001<br>0.0001	0.09	0.02	NS	NS	0.03
Ruminiclostridium 6	0h<br>16h	0.0013<br>0.0007	0.0003<br>0.0002	0.0012<br>0.0009	0.0003<br>0.0002	<0.0001	0.09	NS	NS	0.02
Ruminococcaceae NK4A214 group	0h<br>16h	0.0004<br>0.0002	0.0001<br>0.0001	0.0003<br>0.0002	0.0001<br>0.0001	<0.0001	NS	NS	NS	NS
Ruminococcaceae UCG 004	0h<br>16h	0.0002<br>0.0001	0.0001<br>0.00003	0.0002<br>0.0001	0.0001<br>0.00003	0.001	0.06	NS	NS	NS
Ruminococcaceae UCG 010	0h<br>16h	0.0004<br>0.0002	0.0001<br>0.0001	0.0003<br>0.0002	0.0001<br>0.0001	0.0004	0.07	NS	NS	0.03
Ruminococcus 2	0h<br>16h	0.0017<br>0.0014	0.0005<br>0.0004	0.0015<br>0.0011	0.0003<br>0.0002	<0.0001	NS	NS	NS	0.01

Taxa	Time point	CX		PEG		*P* value				
		Mean	SEM	Mean	SEM	Donor	Tpt	Trt	Trt*Tpt interaction	Donor*Trt*Tpt interaction
Streptococcus	0h	0.00002	0.00001	0.00002	0.00001	0.01	0.06	NS	NS	NS
	16h	0.00005	0.00002	0.00003	0.00001					
Unclassified Christensenellaceae	0h	0.0001	0.00002	0.00003	0.00001	0.002	NS	NS	NS	NS
	16h	0.00004	0.00001	0.00004	0.00001					
Veillonella	0h	0.0009	0.0003	0.0007	0.0002	0.03	NS	NS	NS	0.01
	16h	0.0013	0.0003	0.0009	0.0003					
Victivallis	0h	0.0008	0.0002	0.0004	0.0001	<0.0001	NS	NS	NS	0.01
	16h	0.0005	0.0001	0.0004	0.0001					

Supplementary Table 5. Mean concentration (pg/ml) of pro-inflammatory cytokines after 16h of incubation was decreased in a donor-dependent manner. (A) IL-8 and (B) CXCL-16. n = 8. Different superscripts indicate significantly different means between volunteers within a treatment.

IL-8				*CXCL-16*		
Donor	Treatment		*P* value	Treatment		*P* value
	CX	PEG		CX	PEG	
D1	1189.11 ± 45.47^{ab}	1267.75 ± 19.78^{ab}	NS	15.10 ± 1.27^{a}	13.30 ± 0.29^{a}	NS
D2	1213.53 ± 53.66^{ab}	1354.58 ± 19.95^{ab}	0.03	8.43 ± 0.74^{a}	18.63 ± 2.26^{a}	0.0005
D3	1104.00 ± 39.87^{b}	1150.83 ± 40.06^{b}	NS	8.50 ± 1.62^{a}	11.83 ± 2.14^{a}	NS
D4	1287.64 ± 17.43^{c}	1233.41 ± 7.16^{a}	NS	12.77 ± 0.87^{a}	14.23 ± 2.50^{a}	NS
D5	1164.89 ± 47.33^{ab}	1149.47 ± 42.63^{c}	NS	7.97 ± 0.74^{b}	5.93 ± 0.87^{b}	NS
D6	1284.44 ± 10.35^{a}	1353.36 ± 14.18^{a}	0.05	10.63 ± 0.47^{a}	18.70 ± 2.51^{ac}	0.007
D7	1235.80 ± 35.05^{a}	1268.78 ± 24.55^{a}	NS	26.40 ± 1.47^{c}	17.00 ± 1.72^{c}	0.001
D8	1340.86 ± 27.92^{d}	1268.44 ± 24.33^{a}	0.05	18.70 ± 2.05^{ad}	7.40 ± 0.52^{d}	0.0001

Illumina library preparation and purification

The V5-V6 hypervariable region of the 16S rRNA gene from the cDNA was amplified using primers 341F and 785R. Illumina sequencing adapters and dual-index barcodes were added to the amplicon, using a limited-cycle PCR that included an initial denaturation step at 95 ºC for 3 min, 15 cycles of a denaturation step at 95ºC for 30 s, an annealing step at 55 ºC for 10s, an extension step at 72 ºC for 45 s, and a final extension at 72 ºC for 5 min. Following, a clean-up step was performed using the AMPure XP beads (Beckman-Coulter, Krefeld, Germany) to remove free primers and primer-dimer species from amplicons. A second PCR to attach the specific Illumina multiplexing sequencing primers and index primers, was performed. Thermal cycling included an initial denaturation step at 95 ºC for 3 min, 8 cycles of a denaturation step at 95ºC for 30 s, an annealing step at 55 ºC for 30 s, an extension step at 72 ºC for 30 s, and a final extension at 72 ºC for 5 min.

PCR products were verified by gel electrophoresis, purified using the Promega Wizard PCR clean-up kit (Promega, Madison, WI, USA) following the manufacturer's instructions and quantified with the QuantiFluor dsDNA System kit (Promega, Leiden, The Netherlands). High-throughput amplicon sequencing was performed with the Illumina MiSeq platform according to the manufacturer's guidelines at LGC Genomics GmbH (Berlin, Germany).

Contigs were created by merging paired-end reads based on the Phred quality score (of both reads) heuristic as described by (17) in Mothur v.1.33.3 (18). Contigs were aligned to the SILVA database and filtered from those with (i) ambiguous bases, (ii) more than 10 homopolymers, and (iii) those not corresponding to the targeted region. Chimera removal and operational taxonomic unit (OTU) clustering of the sequencing reads was

performed using UCHIME, with the nearest neighbour clustering algorithm implemented in mothur, at 0.03 distance (19). Phylotype representatives were then generated by clustering at 97% similarity (1 mismatch), with a confidence level of at least 80 with Cyanobacteria, Eukaryota, and Archaea lineages removed. For taxonomic classification, sequence composition of the dataset was compared using the RDP Classifier tool (20), and the RDP trainset version 9. Quality of the sequencing and post-processing pipeline was verified by incorporating mock samples (n = 12 species) in triplicate into the same sequencing run. After examining read counts, if any OTU was not classified up to genus level, the consensus sequence was blasted using the SILVA database (21) to obtain an approximate taxonomic classification. Singletons that remained unclassified were culled.
